# Supplementary material for: Tnfrsf10 Signaling is Required to Maintain the Stem Cell Niche in the Zebrafish Lateral Line
Source: bioRxiv. 2025 Apr 19:2025.04.18.649014. Preprint. [Version 1] doi: 10.1101/2025.04.18.649014 (PMC12190914; doi:10.1101/2025.04.18.649014)
Supplement: 1 [file NIHPP2025.04.18.649014V1-supplement-1.pdf]

bioRxiv preprint doi: https://doi.org/10.1101/2025.04.18.649014; this version posted April 19, 2025. The copyright holder for this preprint (which was not certified by peer review) is the author/funder. This article is a US Government work. It is not subject to copyright under 17 USC 105 and is also made available for use under a CC0 license.

Supplementary Figures

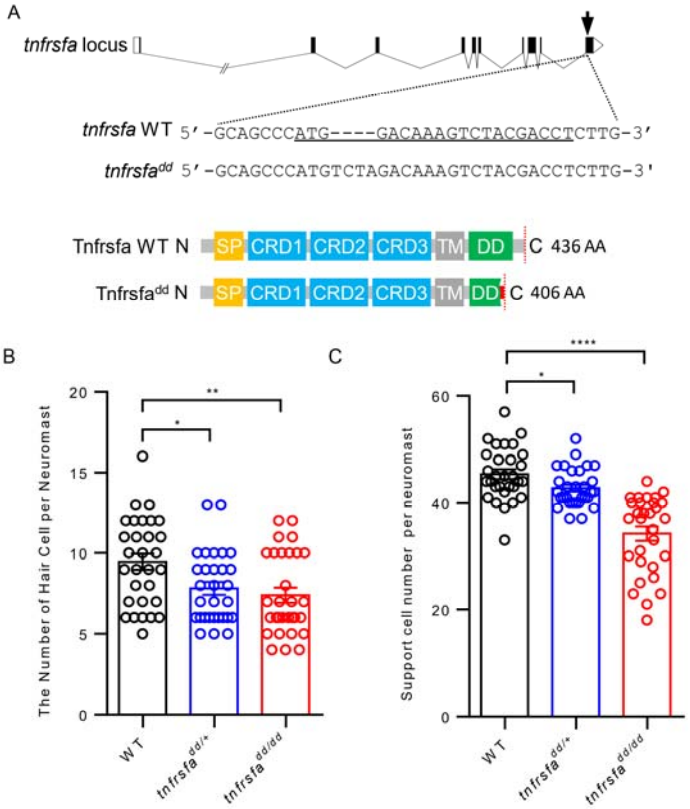

Figure S1. Disrupting the death domain in *tnfrsfa* reduces hair cells and accessory cells in neuromasts is sufficient to inactivate Tnfrsfa. Schematic diagrams showing the generation of *tnfrsfa* mutation by CRISPR/Cas9. Upper panel, the location of gRNA (arrow) in the *tnfrsfa* locus. Middle panel, the sequence surrounding the gRNA of wild-type and mutant *tnfrsfa.* The gRNA target is underlined. Bottom panel, the putative protein structure of wild-type and mutant Tnfrsfa. SP, signal peptide. CRD, cysteine-rich domain. TM, transmembrane domain. DD, death domain. Red box before the stop codon represents missense sequence. **B.** Quantification of hair cells in posterior lateral line neuromasts in wild-type, *tnfrsfa*$^{dd/+}$, and *tnfrsfa*$^{dd/dd}$ larvae at 7 dpf using Yo-Pro-1 staining. Heterozygous mutants appear to have a dominant negative effect. **C.** Quantification of accessory cells in posterior lateral line neuromasts in wild-type, *tnfrsfa*$^{dd/+}$, and *tnfrsfa*$^{dd/dd}$ larvae at 7 dpf by immunostaining with Sox2 antibody.

bioRxiv preprint doi: https://doi.org/10.1101/2025.04.18.649014; this version posted April 19, 2025. The copyright holder for this preprint (which was not certified by peer review) is the author/funder. This article is a US Government work. It is not subject to copyright under 17 USC 105 and is also made available for use under a CC0 license.

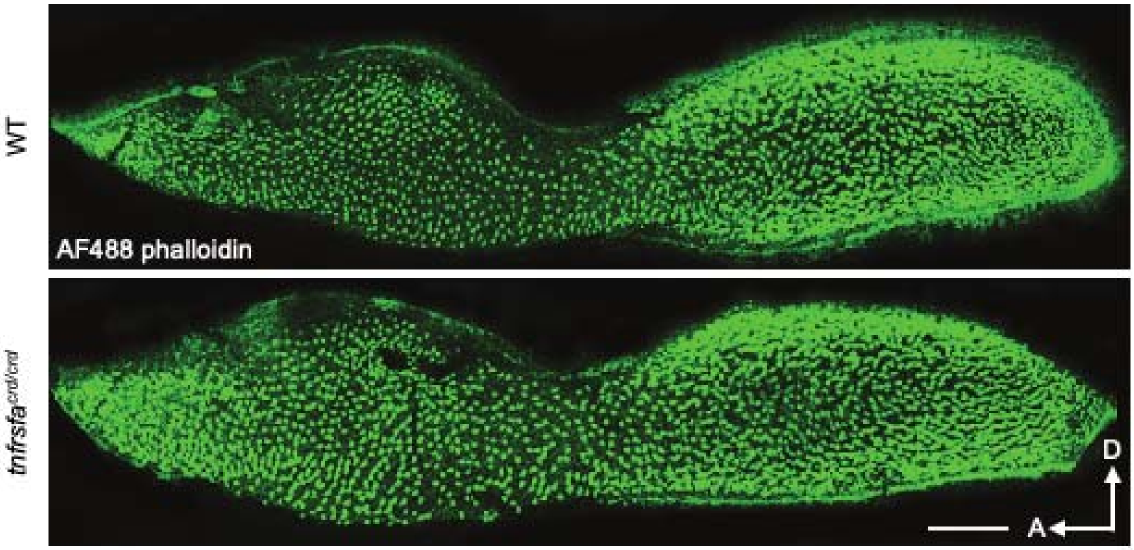

Figure S2. Inner ear development is normal in *tnfrsfa* mutants. Hair cells in the zebrafish inner ear saccule visualized using AF488 phalloidin in *tnfrsfa* mutant and wild-type sibling adults. No significant differences were detected. Scale bar, 100 µm. D, dorsal; A, anterior.

bioRxiv preprint doi: https://doi.org/10.1101/2025.04.18.649014; this version posted April 19, 2025. The copyright holder for this preprint (which was not certified by peer review) is the author/funder. This article is a US Government work. It is not subject to copyright under 17 USC 105 and is also made available for use under a CC0 license.

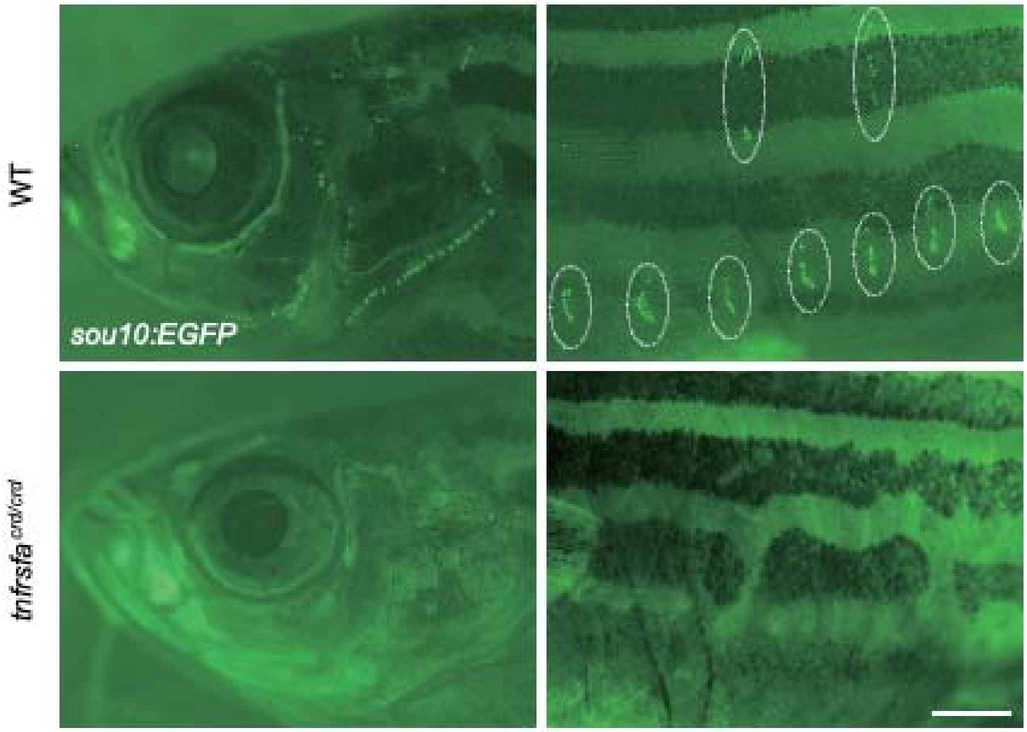

Figure S3. Adult lateral line neuromasts are missing in *tnfrsfa* mutants. Live imaging of support cells in *tnfrsfa*^{crd} mutant and wild-type sibling adults at 3 mpf visualized by the *Tg(sou10:EGFP)* transgenic reporter line. White dashed circles outline superficial neuromast stitches. Scale bar, 100 µm.

bioRxiv preprint doi: https://doi.org/10.1101/2025.04.18.649014; this version posted April 19, 2025. The copyright holder for this preprint (which was not certified by peer review) is the author/funder. This article is a US Government work. It is not subject to copyright under 17 USC 105 and is also made available for use under a CC0 license.

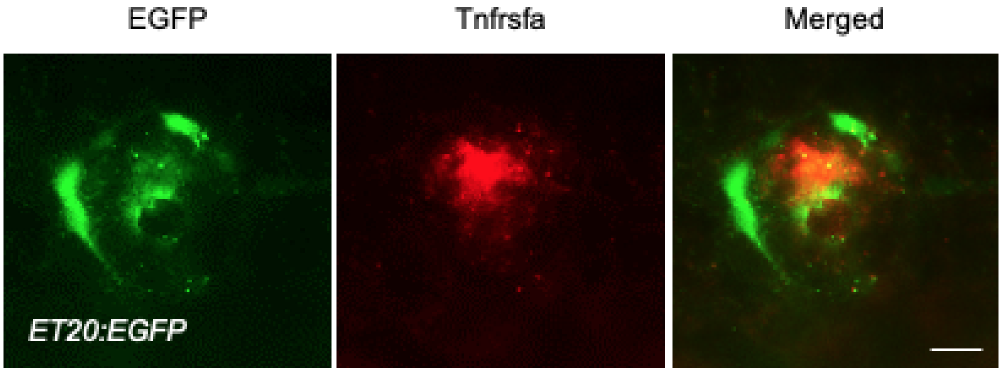

Figure S4. Tnfrsfa protein is strongly expressed in the dorsal support cells. Immunostaining of Tnfrsfa (red) and EGFP (green) in a *Tg(ET20:EGFP)* neuromast. No significant expression was detected in the GFP-positive cells. Scale bar, 10 µm.

bioRxiv preprint doi: https://doi.org/10.1101/2025.04.18.649014; this version posted April 19, 2025. The copyright holder for this preprint (which was not certified by peer review) is the author/funder. This article is a US Government work. It is not subject to copyright under 17 USC 105 and is also made available for use under a CC0 license.

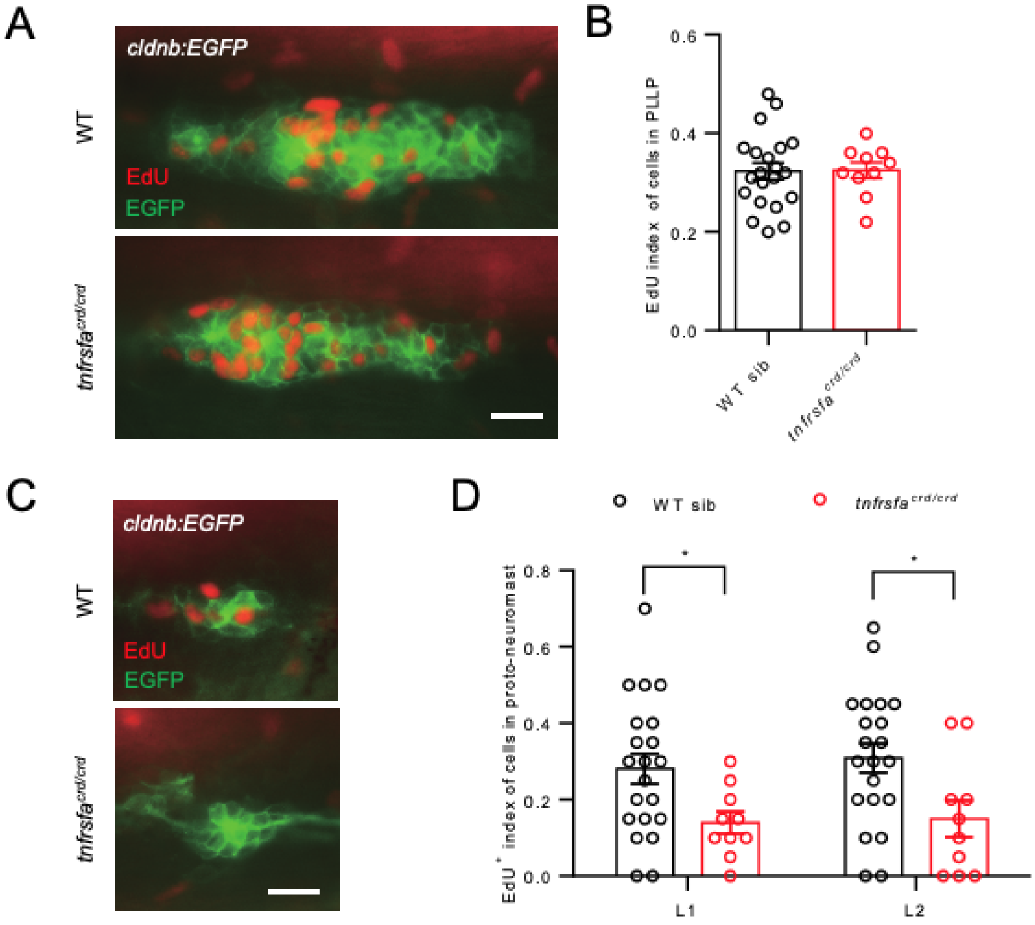

Figure S5. *tnfrsfa* plays a crucial role in cell proliferation in posterior lateral line proto-neuromasts. A, C. 2 h EdU incorporation in posterior lateral line primordium (**A**) and proto-neuromasts (**C**) visualized by *Tg(cldnb:EGFP)* at 30 hpf in wild-type and *tnfrsfa* mutant larvae. EGFP was detected by immunostaining with EGFP antibodies (green). Scale bar, 20 µm. **B, D.** Quantification of EdU indexes of posterior lateral line primordium (**B**) and proto-neuromasts (**D**) in wild-type and *tnfrsfa* mutant larvae in the L1 and L2 protoneuromasts. *, $p < 0.05$.

bioRxiv preprint doi: https://doi.org/10.1101/2025.04.18.649014; this version posted April 19, 2025. The copyright holder for this preprint (which was not certified by peer review) is the author/funder. This article is a US Government work. It is not subject to copyright under 17 USC 105 and is also made available for use under a CC0 license.

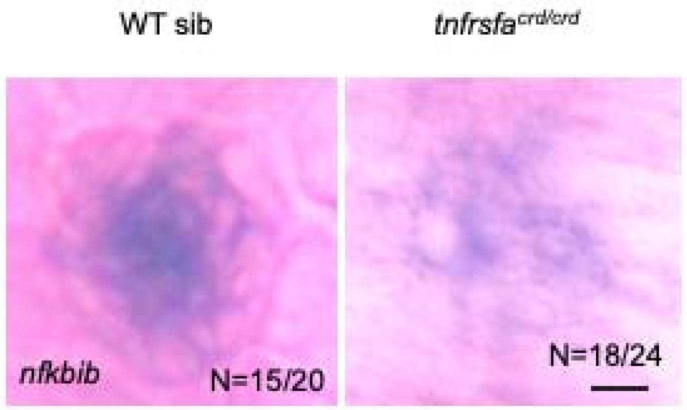

Figure S6. Tnfrsfa activates targets downstream of NF-κB signaling in the support cells.

Whole-mount *in situ* hybridization of *nfkbib* in neuromasts of wild-type and *tnfrsfa* mutant larvae. Scale bar, 10 µm.

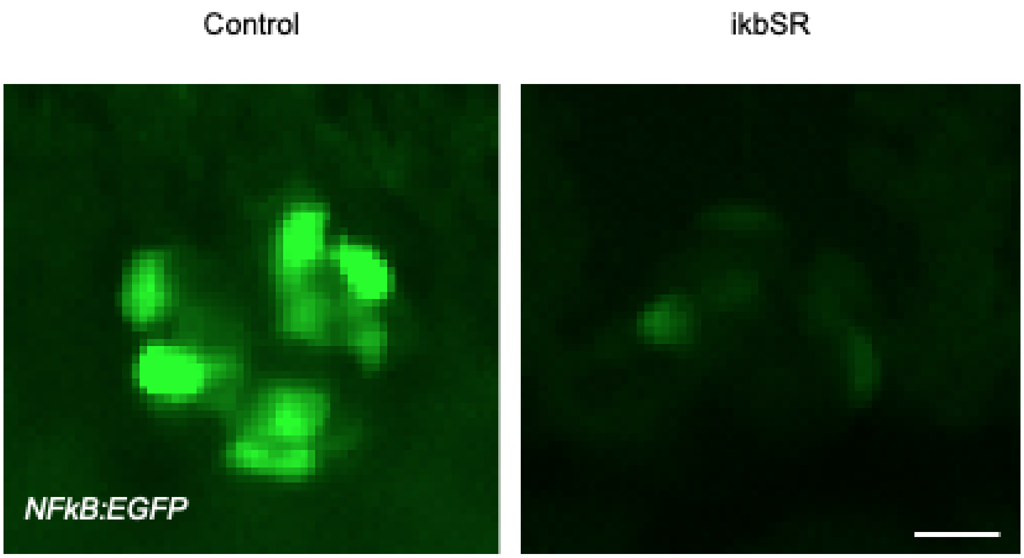

Figure S7. Overexpression of ikbSR inhibits NF-κB signaling in neuromast. Live imaging of neuromast from *Tg(NFkB:EGFP)* (Control) and *Tg(NFkB:EGFP); Tg(hsp70l:ikbSR-mcherry)* (ikBSR) larvae at 5 dpf following heat-shock treatment for 2 days. Scale bar, 10 µm.

bioRxiv preprint doi: https://doi.org/10.1101/2025.04.18.649014; this version posted April 19, 2025. The copyright holder for this preprint (which was not certified by peer review) is the author/funder. This article is a US Government work. It is not subject to copyright under 17 USC 105 and is also made available for use under a CC0 license.

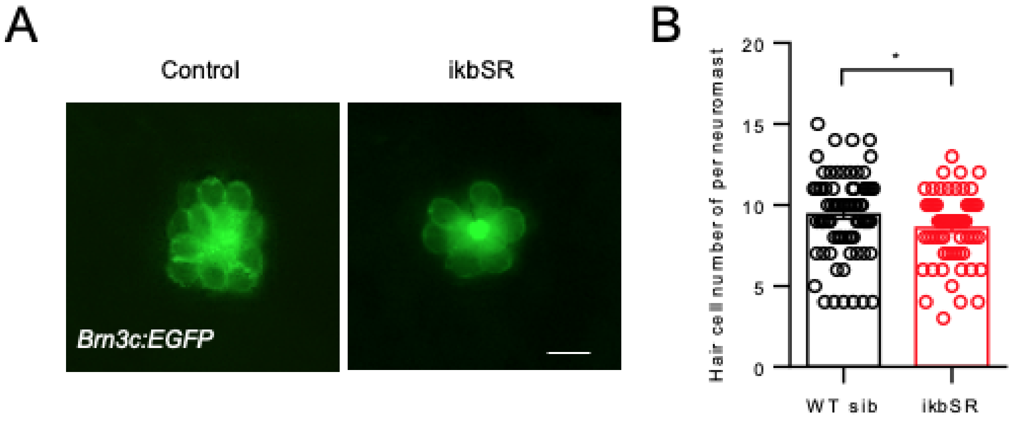

1044

1045 **Figure S8. Overexpression of ikBSR reduces hair cells in posterior lateral line neuromasts. A.**

1046 The live imaging of hair cells in neuromast from *Tg(Brn3:EGFP)* (Control) and *Tg(Brn3:EGFP);*

1047 *Tg(hsp70l:ikbSR-mcherry)* (ikBSR). **B.** Quantification of hair cells in posterior lateral line

1048 neuromasts from *Tg(Brn3:EGFP)* (Control) and *Tg(Brn3:EGFP); Tg(hsp70l:ikbSR-mcherry)* (ikBSR).

1049 Scale bar, 10 µm.

1050

bioRxiv preprint doi: https://doi.org/10.1101/2025.04.18.649014; this version posted April 19, 2025. The copyright holder for this preprint (which was not certified by peer review) is the author/funder. This article is a US Government work. It is not subject to copyright under 17 USC 105 and is also made available for use under a CC0 license.

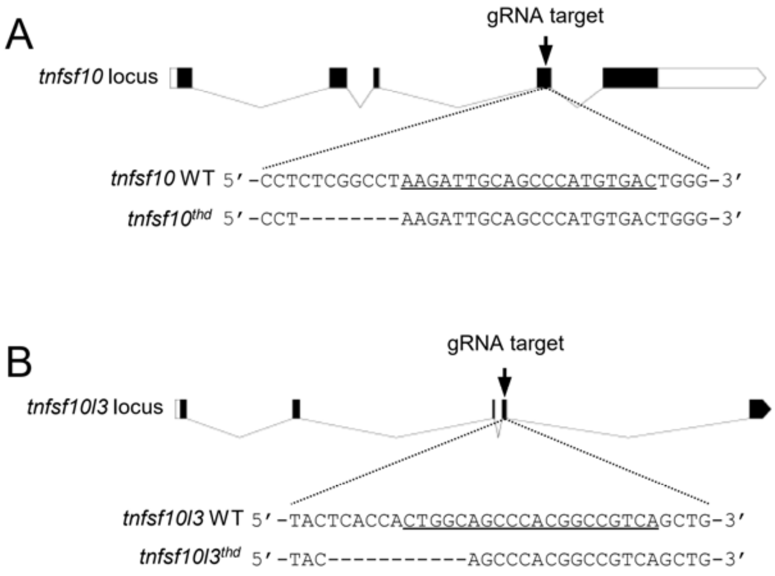

Figure S9. Targeted knockdown of the Tnfrsfa ligands. Schematic diagrams of generating *tnfsf10* (**A**) and *tnfsf10l3* (**B**) mutations generated by CRISPR/Cas9. Upper panel, the location of gRNA (arrow) in gene locus. Middle panel, the sequence around gRNA of wild-type and mutant. The gRNA target is underlined.

bioRxiv preprint doi: https://doi.org/10.1101/2025.04.18.649014; this version posted April 19, 2025. The copyright holder for this preprint (which was not certified by peer review) is the author/funder. This article is a US Government work. It is not subject to copyright under 17 USC 105 and is also made available for use under a CC0 license.

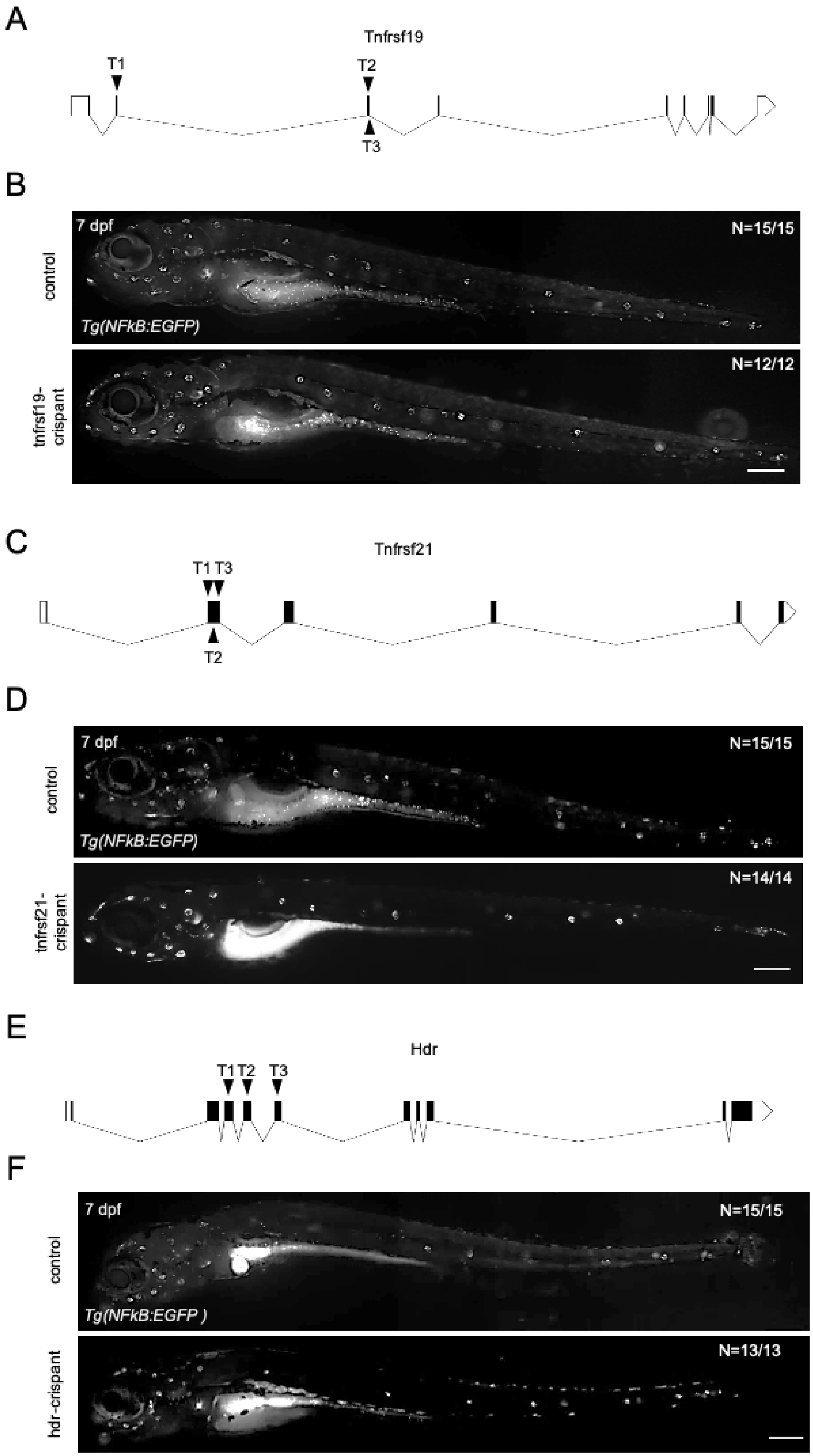

bioRxiv preprint doi: https://doi.org/10.1101/2025.04.18.649014; this version posted April 19, 2025. The copyright holder for this preprint (which was not certified by peer review) is the author/funder. This article is a US Government work. It is not subject to copyright under 17 USC 105 and is also made available for use under a CC0 license.

Figure S10. The impact of *tnfrsf19*, *tnfrsf21* or *hdr* mutations on NF-κB signaling in zebrafish lateral line neuromasts. A, C, and **E:** Schematic diagram of generating *tnfrsf19-*, *tnfrsf21-* and *hdr-crispants* using three gRNAs. **B, D,** and **F:** The fluorescence of *Tg(NFkB:EGFP)* in control and *tnfrsf19-*, *tnfrsf21-* and *hdr-crispants.* No major effects were seen. Scale bar: 200 µm.

bioRxiv preprint doi: https://doi.org/10.1101/2025.04.18.649014; this version posted April 19, 2025. The copyright holder for this preprint (which was not certified by peer review) is the author/funder. This article is a US Government work. It is not subject to copyright under 17 USC 105 and is also made available for use under a CC0 license.

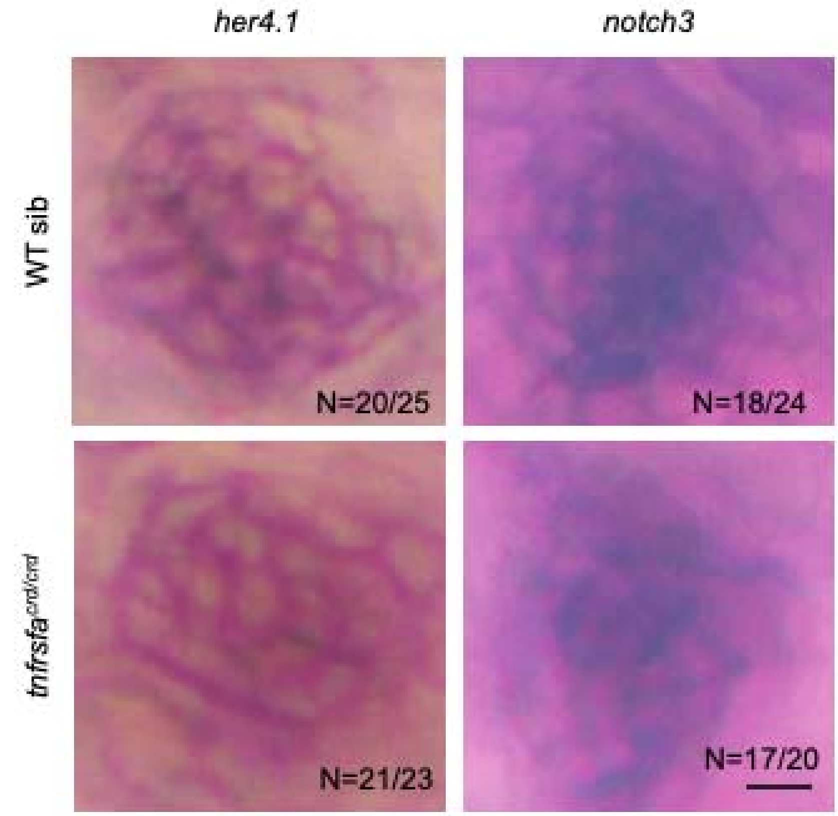

Figure S11. Mutations in tnfrsfa do not impact Notch activity. Whole-mount *in situ* hybridization showing the expression of *her4.1* and *notch3* in wild-type and *tnfrsfa* mutant zebrafish neuromasts. Scale bar, 10 µm.
